# Transcatheter Aortic Valve Implantation for Severe Bicuspid Aortic Stenosis – 2 Years Follow up Experience From India

**DOI:** 10.3389/fcvm.2022.817705

**Published:** 2022-07-28

**Authors:** Vijay Kumar, G. Sengottuvelu, Vivudh P. Singh, Vishal Rastogi, Ashok Seth

**Affiliations:** ^1^Fortis Escorts Heart Institute, New Delhi, India; ^2^Apollo Hospitals, Chennai, India

**Keywords:** aortic stenosis, bicuspid aortic valve, bicuspid aortic stenosis, Indian population, TAVI – transcatheter aortic valve implantation

## Abstract

**Background:**

Transcatheter aortic valve implantation (TAVI) is challenging in bicuspid aortic valve (BAV) anatomy. The patients are young, morphological phenotypes are many, calcium burden is high and there are technical challenges for best outcomes. Observational studies and registries are available with favorable data and experiences from around the world sharing methodologies and algorithms for sizing and implantation. We, therefore, analysed our data of procedural and in-hospital outcomes of TAVI in Bicuspid Aortic Valve cases performed at two high volume centres in India and their follow up for two years.

**Methods and Results:**

The data were collated and analysed from two centres (Fortis Escorts Heart Institute, New Delhi and Apollo Hospitals, Chennai) in India for patients who underwent TAVI in a BAV anatomy. It included a total of 70 cases from 2 centres. All symptomatic severe AS patients more than and equal to 65 years having bicuspid anatomy were included in the study irrespective of their STS score. Patients under 65 years of age were advised TAVI only if they were at high risk for open heart surgery. These patients were followed for a period of 2 years and the data were analysed. Pre TAVI imaging tools utilised were 2D echo, transthoracic echocardiography (TTE), trans oesophageal echocardiography (TEE), and ECG gated multi slice CT (MSCT) scan imaging. MSCT was utilised for confirmation of the anatomy and classifying the morphological type of valve, measuring, and evaluating all anatomic determinants of aortic root complex for planning the procedure and choice of the valve and its size. Sizing in balloon expanding valve (BEV) and self-expanding valve sizing (SEV) were based primarily on annulus area and perimeter, respectively. The SEV used in our study were the Core Valve and Evolut R (Medtronic, United States) and the BEVs included Sapien3 (Edwards Lifesciences, United States) and Myval (Meril Lifesciences, India). The BAV cohort constituted 24.4% of the total 287 TAVI cases, followed up for 2 years. The mean age of these patients was 72 years. The incidence of male patients was 68.57% and female patients was 31.4%. The Sievers type 1 included 78.5%, type 0 were 21.4% of the cases and there was no case of type 2 in the study. The procedural success was to the tune of 98%. Patients with normal left ventricular ejection fraction (LVEF) improved their symptoms class after TAVI and remained so at 2 years follow up. The poor LVEF subset of patients did not have heart failure admissions and also had improvement in their symptom status. The peak-to-peak aortic valve gradient decreased to 0 mmHg at the end of the procedure in most of the cases. The mean pressure gradient (PG) across the new valve ranged between 0 and 15 mmHg and the aortic valve area (AVA) was close to 2 cm^2^. These numbers were consistent at 2 years follow up. Significant paravalvular leak (PVL) 24.28% was seen immediately after deployment of the valve in heavily calcified anatomy but it reduced to mild or trivial PVL after post-dilation and one patient needed a second valve to treat PVL. No patient had more than mild PVL with either type of valve at the end of the procedure. Permanent pacemaker implantation (PPI) was required in 11.4% of the patients within 24 h to 7 days of the procedure. No one needed a PPI in the 2 year follow up. Coronary occlusion did not happen to any patient. No patient had a disabling stroke. Non-disabling stroke was seen in 10% of cases and mostly in the first week or 30 days of the procedure and the incidence was more with BEV (14%) as compared to SEV (8%). There was one case of valve embolisation after 24 h of the procedure, which needed a surgical valve replacement. There was no case of annular injury or injury to other parts of the aortic root complex. Two cases had access vessel (femoral artery) thrombosis at end of the procedure and a third patient had proglide related residual stenosis. Two cases had acute kidney injury and needed dialysis. There was no major bleeding complication in any patient. Peri procedural mortality occurred in two patients. Valve thrombosis was seen in one patient after 3 months, which was treated with oral anticoagulation. Valve degeneration and failure or infective endocarditis were not seen in any patient.

**Conclusion:**

The patients with BAV stenosis who underwent TAVI in this study had good procedural success rates and clinical outcomes. The haemodynamics achieved with both SEV and BEV were good at 2 years. The rates of PVL, PPI, and stroke are similar to that of many other studies and registries. PPI rate and non-disabling stroke incidence appear to be higher similar to many studies done. There was no case of coronary occlusion in the study. Meticulous CT analysis of the aortic root complex, selection of appropriate type and size of the valve, and best implantation practices along with cerebral protection will probably be the key to safer and more successful TAVI in this population.

## Introduction

Transcatheter aortic valve implantation (TAVI) has become an established treatment for the tricuspid aortic valve in high and intermediate-risk patients with good outcomes and long follow up data. Favourable data for low-risk patients are also in abundance now for the tricuspid valve population (1–5). On the other hand, in bicuspid aortic valve (BAV) disease, the inherent anatomical challenges (6, 7) make TAVI in this subset not easy and straightforward. More data will be required to support the therapy, especially for low-risk patients who are younger and cannot afford to have residual significant gradients, patient prosthesis mismatch, any significant paravalvular leak (PVL), lifelong implantation of a pacemaker, coronary ostia occlusion, and difficult future coronary interventions. This study shares the results of TAVI in India for patients with BAV anatomy.

## Materials and Methods

### Study Design and Patient Population

We collected data for 287 consecutive patients who underwent TAVI at Fortis Escorts Heart Institute, New Delhi and Apollo, Chennai, India between the year 2012 and 2018. The cohort of BAV anatomy who underwent TAVI included a total of 70 patients. TAVI was chosen for symptomatic severe AS patients who were 65 years and above with a low, intermediate, or high STS risk score (8). Those less than 65 years of age were advised of TAVI if they were high risk cases for SAVR. The BAV could be of any Sievers type (9) of morphology. Exclusion criteria constituted those of age less than 65 years, prohibitive STS risk score (8), patients with other significant valve pathology, severe LV dysfunction left ventricular ejection fraction (LVEF) <20%, the aortic annulus size was out of the range of size of devices available, and if the risk of coronary occlusion was a concern as assessed by the multi slice CT (MSCT) analysis. Patients who had survival of less than a year due to some terminal illnesses were not included in the study. Rheumatic heart disease or multivalvular pathology, pure aortic regurgitation, and valve in valve procedures were also excluded. TAVI was performed under conscious sedation for the majority of cases. The transfemoral route was used for all the cases. The self-expanding valve (SEV) used was Core Valve and Evolut R (Medtronic, United States); and the balloon expanding valve (BEV) used in our study were Myval (Meril Lifesciences, India) and Sapien3 (Edwards Lifesciences, United States). The standard implantation techniques for each type of valve were followed step by step (10–12). The success of the TAVI procedure, complications and clinical outcomes were all defined as per the Valve Academic Research Consortium 2 and 3 (VARC-2 and 3) consensus (13, 14). Patients were put on 75 mg of clopidogrel and aspirin for 3 months and then lifelong aspirin after TAVI. Anticoagulation was on board if there was an indication and or if there was a conformation of thrombus formation by CT scan on the valves in their follow up period. The study was approved by the Internal Review Board and Ethics Committee approval was not required.

### Follow up and Data Collection

The patients were followed up at 7 days, 1 month, then annually for 2 years after the TAVI. The majority of patients visited the primary centre, while the others were followed telephonically, and their echo and ECG records performed at another centre were retrieved and added to our database. At each visit, the patient’s NYHA functional class was evaluated, and an ECG and standard 2D echo was performed. All clinical events were recorded. Major adverse cardiovascular events were defined as death stroke and myocardial infarction as previously described.

### Imaging Methods

Patients underwent a standard screening echocardiogram and contrast enhanced ECG gated multi detector computed tomography (MDCT) before the procedure by the standard of imaging practised for pre and post TAVI work up (15–19). The CT analysis was done by a dedicated 3mensio medical imaging pie medical imaging software. Pre TAVI-CT focussed on confirmation of the bicuspid anatomy, morphological types, calcium score, topography of calcium causing injury to the annulus, LVOT, and coronary ostia occlusion factors. The sizing of the valve was carefully decided mainly based on the size of the annulus, supra-annulus, and LVOT dimensions. Area measurements of annulus and LVOT were considered in the case of BEV. The perimeter of the annulus, LVOT, supra-annular measurements at 4.5 and 8 mm above the annulus were measured for SEV. Intercommissural distance was also an important parameter in choosing the valve size in SEV (16). Contemporary sizing algorithms like CASPER (Calcium Algorithm Sizing for bicusPid Evaluation with Raphe) and **LIRA** (**L**evel of **I**mplantation **RA**phe Annulus method-for Raphe type BAV) were also utilised to decide the size of the valve (20–24). Measurements of STJ, SOV, calcium burden, and distribution, coronary ostia occlusive factors were also considered while sizing the valve (25). BEVs were upsized by 5–10% and SEVs were upsized by 15–25%.

### Statistical Methods

The statistical analysis consisted of patient demography, frequency calculation in terms of all baseline characteristics such as age, weight, sex, etc. All continuous variables were analysed using the Student’s *t*-test at 95% CI to evaluate the significance for various parameters before, immediately after 7 days later, 1 and 2 years following the TAVI procedure. The non-parametric parameters such as calcification, NYHA functional class, the severity of PVL were estimated using the Chi-square test. Data analysis and interpretation were performed with Stata software ReDEA Institute of Data Science (RIDS). Bartlett’s test for equal variance and pairwise tests of difference of means by Games and Howell were applied to evaluate the statistical significance we accounted for the non-homogeneity of variance present in the comparison groups for the pre and post-procedural periods mean pressure gradient (PG) and aortic valve area (AVA) (26).

### Study Outcomes

Device implantation was defined as successful vascular access, delivery, and deployment of a single device in the proper anatomic location, the appropriate performance of the THV and retrieval of the delivery system (VARC-2). Valve performance was assessed by measuring mean gradient across the transcatheter heart valve, aortic valve area (AVA) of the THV, and the absence of significant PVL after TAVI at 7 days, annually and 2 years. The need for a permanent pacemaker after the procedure or follow up was also collected into the database at 2 years follow up. THV thrombosis and degeneration was recorded over the two years. Other study outcomes included in-hospital mortality, stroke, VARC-2 major bleeding, acute kidney injury, and vascular complications, which were recorded for analysis. LV systolic function improvement from baseline was also analysed at 2 years. The functional class of the patients was collected from the data for 2 years.

## The Results and Analysis

The baseline characteristics of bicuspid aortic valve patients in our study have been summarized in Table 1. Table 2 depicts the procedural characteristics and outcomes of TAVI in the study. The total TAVI cases from 2 centres in India included a total of 287 patients with symptomatic severe aortic stenosis followed up for 2 year period. A total of 70 patients (24.4%) constituted the bicuspid aortic population who underwent TAVI. The mean age of patients in our study was 72 years. The majority of patients were in the sixth and seventh decade of life. Male patients constituted 68.57% and female patients were 31.43% of the study population. The PROM STS score consisted of the majority of patients in the intermediate (47%) or low risk (32%) group and high-risk patients accounted for 20%. The patients belonged mostly to NYHA II–III (70–80%) and 20–30% belonged to NYHA IV. Dyspnoea was the major presenting symptom for most patients.

**TABLE 1:** Base line characteristics of bicuspid aortic valve patients in the study.

Total patients	70
Male: female	48:32
Mean age (years)	72 (±8.49)
Mean weight (in kg)	67.54 (±11.17)
Height (in cm)	161.42 (±8.01)
BMI	25.14 (±6.23)
**PROM STS score**	
High (0–4)	20%
Intermediate 4–8	47.14%
Low risk ≤	32.86%
Mean STS score	6.00 (±6.54)
Baseline LVEF (%)	50.6 (±13.66)
**Pre TAVI-NYHA class**	
I	1.4%
II	37%
III	35.7%
IV	25.7%
**Sievers type**	
Type 1	78.5%
Type 0	21.4%
**Number of raphe**	
Type 0	No raphe
Type 1	98% had one raphe
**Pattern of cuspal fusion**	
Type 0	AP 46.6% lateral 53%
Type 1	RCC LCC 96% RCC NCC 3.6%
Severity of calcium in raphe	50% mild 12–27% moderate 18–37% severe
Right coronary ostia height (mm)	16.9 (±3.3)
Left coronary ostia height (mm)	14.75 (±4.09)
Aortic valve calcification	70%-severe 30%-moderate
Aortic root dilation	85.7% ≤40 mm 14.3% ≥41 mm

**TABLE 2:** Procedural characteristics and outcomes.

**Anaesthesia used**	
Local	85.51% (59)
General	14.49% (10)
**Access vessel (transfemoral)**	100% (69)
**Predilation of native valve**	100% (69)
**Valve type used**	
BEV	30% (21)
SEV	70% (49)
**Paravalvular leak immediately at end of implantation**	
Mild	68.12% (47)
Moderate	17.39% (12)
Severe	5.8% (4)
Trace	2.9% (2)
Trivial	1.45% (1)
None	4.35% (3)
**Post-dilation done to reduce paravalvular leak**	
No	76.81% (53)
Yes	23.19% (16)
**Paravalvular leak at 7 days**	
Mild	11.59% (8)
None	76.81% (53)
Trace	11.59% (8)
**Paravalvular leak at 2 years**	
Mild	11.76% (8)
None	77.94% (53)
Trace	10.29% (7)
**Average of mean pressure gradient at end of procedure (mmHg)**	8.5
**Average of mean pressure gradient at 2 years after procedure (mmHg)**	8.4
**Average of aortic valve area pre TAVI (cm^2^)**	0.54
**Average of aortic valve area 2 years after procedure (cm^2^)**	2.03
**Disabling stroke In first 30 days after procedure**	
No	100% (70)
Yes	0% (0)
**Non-disabling stroke In first 30 days after procedure**	
No	90% (63)
Yes	10% (7)
**Disabling stroke in 2 years after procedure**	
No	100% (70)
Yes	0% (0)
**Non-disabling stroke 2 years after procedure**	
No	100% (70)
Yes	0% (0)
**Average of Echo LVEF % baseline**	50.6
**Average of Echo LVEF % 2 years after procedure**	53.2
**Pre-TAVI dyspnoea NYHA class**	
I	1.43% (1)
II	37.14% (26)
III	35.71% (25)
IV	25.71% (18)
**Post-TAVI dyspnoea NYHA class 2 years after procedure**	
I	95.71% (67)
II	4.29% (3)
**Non-disabling stroke after 30 days with**	
*Balloon expanding valve*	
No	85.71% (18)
Yes	14.29% (3)
*Self expanding valve*	
No	91.84% (45)
Yes	8.16 % (4)
**Complete heart block needing permanent pacemaker implantation by 30 days**	
*Balloon expanding valve*	
No	95.3% (20)
Yes	4.7% (1)
*Self expanding valve*	
No	83.67% (41)
Yes	16.32% (8)
**Vascular complications**	
None	92.86% (65)
Proglide mediated stenosis	1.43% (1)
Thrombotic occlusion	5.72% (4)
**PVL immediately at end of implantation**	
*Balloon expanding valve*	
Mild	60% (12)
Moderate	20% (4)
Severe	0% (0)
Trace	5% (1)
Trivial	0% (0)
None	15% (3)
*Self expanding valve*	
Mild	71.43% (35)
Moderate	16.33% (8)
Severe	8.16% (4)
Trace	2.04% (1)
Trivial	2.04% (1)
None	0% (0)
**PVL at 7 days with**	
*Balloon expanding valve*	
Mild	5% (1)
None	80% (16)
Trace	15% (3)
*Self expanding valve*	
Mild	14.29% (7)
None	75.51% (37)
Trace	10.20% (5)
**PVL at 2 years**	
*Balloon expanding valve*	
Mild	5% (1)
None	80% (16)
Trace	15% (3)
*Self expanding valve*	
Mild	14.58% (7)
None	77.08% (37)
Trace	8.33% (4)
**Acute kidney injury**	
No	95.71% (67)
Yes	4.29% (3)
**THV thrombosis at 2 years**	
No	98.57% (69)
Yes	1.43% (1)
**THV degenration at 2 years**	
No	100% (70)
Yes	0% (0)
**Procedural mortality**	None
**Mortality at 7 days after procedure**	None
**Mortality at 30 days after procedure**	2.86% (2)
**Mortality at 2 years after procedure**	2.86% (2)

Sievers bicuspid valve class of distribution consisted of type 1 (78%), type 0 (21%), and there was no type 2 patient in our study. The number of raphe present in type 0 was zero, one in 98% of type 1 Sievers. There was a very rudimentary raphe rest of 2% for type 1 patients. The commonest cuspal fusion was between the right coronary cusp (RCC) and left coronary cusp (LCC) in type 1. The type 0 had equal types of cusps anterior–posterior and lateral cusps. The distribution of calcium in the raphe was mostly moderate or severe though some patients had a milder degree of calcium in raphe. There was not much difference in male: female and Sievers types of bicuspid valve. The aortic valve calcification was assessed by MSCT and consisted mostly of moderate and severe calcification. A dilated ascending aorta, which is one of the manifestations of aortopathy in BAV patients, was seen in about 14% of patients and the size was between 40 and 51 mm.

The composite end points as given in VARC-2 and 3 consensuses were analysed for all 70 patients (13, 14). **Procedural success rate** was 98.2%. Mortality of two patients with numerous comorbidities occurred in our cohort related to chest infection, pneumonia, and sepsis with acute kidney injury in the immediate post procedure period. **The type of valves used** were self expanding in 70% of the cases whereas the balloon expanding platform was used in 30% of cases. The **peak-to-peak gradient** decreased from preprocedural values to less than 15 mmHg in the majority of the patients. There was a need to post dilate in a small percentage of cases where the residual gradients were more than 15 mmHg or more than mild PVL was seen after deployment.

There was a significant **PVL** in close to 20–30% of patients immediately after the procedure who needed post dilation mostly because of the unexpanded frame and the leak reduced to trivial or mild requiring no further action. At the end of the procedure, there was no PVL in 80–85%, mild PVL in 5–7 and 5–20% of patients had trace PVL. There was no statistically significant difference in the degree of PVL between the two groups who had SEV and BEV. The PVL was similar at the end of 2 years whether a BEV or SEV was implanted. Calcium in raphe and valve leads to significant PVL after deployment of the valve. **The mean gradient** of the valve after the procedure also decreased to less than 15–20 mmHg in 100% of the patients as seen at 7 days, 30 days and the mean gradient continued to be mostly less than 15 mmHg–20 mmHg at 2 years after TAVI. **The mean AVA** similarly increased from <1.0 cm^2^ to >1.3–2.0 cm^2^ in the majority of patients in the same timeline of 2 years after TAVI. Whether this decrease in mean pressure gradient and increase in aortic valve area was statistically significant or not was further analysed by pairwise tests of difference of means by the method described by Games and Howell where adjustment for the unequal variances is done in their formulas to calculate the size of 95% confidence intervals. The average AVA continued to maintain the immediate post procedural values. The PG across the THV remained the same as the post procedure in most of the patients.

**The LVEF** improved from pre TAVI level to normal or near normal function (LVEF-55–60%). Poor LV systolic function patients improved their function marginally, but the sample size was small in our study and hence analysis was not possible. They had improvement in symptoms and did not have heart failure related admission in the 2 year follow up period. **Improvement in symptoms and functional class** changed and remained the same at 2 years. Most of the patients were in class I at 2 years after TAVI. A valve wise analysis also revealed a similar improvement in the class of patient’s symptom status. **Stroke** occurred in 10% of the patients in post procedural period (24 h to 30 days) and was of a non-disabling nature. They all recovered their neurological deficits in 24 h to 4 weeks period. The spectrum of neurological deficit was weakness of hand grip, slurring of speech, aphasia, monoparesis, and or psychiatric manifestation like delirium and confusion. The MRI revealed showers of microemboli in these patients. There was no dense stroke in any case. A valve wise analysis of stroke was performed, which revealed the incidence to be more with BEVs (14.2%) as compared to the 8.1% of patients who had self-expanding valve implantation done. No patient had a stroke beyond 30 days to 2 years of follow up. **High degree conduction system block** did not occur in the majority of patients. The pacemaker implantation rate was 16% with SEV and 4% with BEVs. There was no **life-threatening** bleeding in any case though some patients received transfusions who had low baseline haemoglobin. **The vascular complications** that occurred in three patients were thrombus formation of the access vessel in two cases: one was managed with ballooning and the other case needed stenting. One patient had proglide related stenosis that was managed by gentle balloon dilation. **All-cause mortality was none at 1 and 2 years** follow up of our patients. **THV valve degeneration and failure at 2 years follow up** was not seen in any case nor the need for balloon valvuloplasty, TAV in TAV or surgical valve replacement was required. Lastly, there were no cases of infective endocarditis in our patients within 2 years follow up. One patient showed increased mean gradient of 40 mmHg across the THV in follow up at 6 months, MSCT showed valvar thrombosis. It was successfully treated by oral anticoagulation.

## Discussion

Data regarding the epidemiology of valvular heart disease in India remains scant because of a lack of resources and the maintenance of poor medical records. A single centre study by Manjunath et al. from a high-volume centre in India showed isolated aortic stenosis as the third most common (7.3%) valve lesion in an adult population and degenerative calcific as the most common cause (65%) followed by BAV (33.9%) (27). Rheumatic heart disease contributes to 1.1%. Isolated AS was more common in male patients. In the study again 65.3% had pure AS, 21.9% had pure AR and 12.8% had combined lesion (27).

The first clinical experience of TAVI in India was in 2012 in an octogenarian lady with a previous history of CABG and a porcelain aorta with severe AS that was left unoperated for 12 years and became the cause of her recurrent heart failure admissions (28). TAVI procedure is currently done in 30 centres across India out of which 7 centres cater to the maximum cases (29). Since its introduction, the technology has rapidly expanded and seems on its way to having achieved an all-risk indication and both bicuspid and tricuspid populations are inclusive. Apart from the anatomical factors characteristics of BAV, the important challenges of TAVI in Indian population are cost and reimbursement policy, regulatory body approval, the learning curve and acquiring proficiency by the operators performing the TAVI procedure (29). In a young BAV population, it is a difficult decision for both the physician and the patient to choose TAVI over SAVR as per the present evidence and challenges of this therapy. In a study from India by Sahu et al. (30) a unique observation was made that 60% of TAVI patients are less than 60 years of age and they may not call for TAVI, age is an important determinant for TAVI. It thus has thus important implications for the penetration of TAVI in the Indian subcontinent unless robust evidence is established (30). Our study shows a similar mean age group of patients undergoing TAVI for BAV with severe stenosis as that in the other studies. Male predominance has been found to be there similar to other studies. Type 1 and type 0 are the commonest morphological types and the calcium score of the valves has been moderate to severe in our population.

Outcomes of the early experiences with TAVI in BAV patients were not encouraging in the world data. In the first TAVI series in 2010 (11), the rate of periprocedural complications was high with 13–34% equal to or greater than moderate PVL, 13–43% needed permanent pacemaker implantation (PPI), and 1 year mortality was 4–18% (31). In 2007 Yoon et al. reported a PVL of 10.4%, PPI was 14.7% (32). The STS/ACC TVT registry with all generations of valves showed a PVL of 4.7% at 1 year and the 1 year hazard of stroke (HR, 1.14 (95% CI 0.94–1.39) in the BAV arm (33). Perlman et al. first described a series of 51 patients without any or equal PVL in whom Sapien3 was used (32). The STS/ACC TVT registry also showed better outcomes with the newer generation of valves with a moderate PVL of 3.2%, stroke at 1 year of 3.4%, and 9.1% had PPI. Forrest et al. reported a 15.4% PPI rate with Evolut R/pro and a 3.9% stroke rate at 1 year. Overall, the short-term outcomes improved dramatically with the new generation of valves (34). Waksman has reported no death and no disabling stroke in 61 low risk BAV patients at 30 days, The rate of PPI was 13% and moderate PVL was just 1.6% (35). Similarly the low-risk bicuspid study had 1 death and 1 case of disabling stroke, and PPI was 15% (36). The BIVOLUT X study also showed promising results for TAVI in BAV patients with no PVL and excellent haemodynamic outcomes.

The results of our study are comparable with the aforementioned studies. The patients had a mean STS risk score of 6% and a majority of them had a newer generation of THV implanted. The outcomes of this study are comparable with **other observational studies where low to intermediate risk patients constituted the major percentage of patients**. The calcium burden was mild to moderate in most patients. Type 1 Sievers were the most common variant. SEV were more used than the BEV. Device success, valve performance and clinical outcomes are matched.

The results of our study demonstrate good clinical outcomes among all the patients who underwent TAVI across all risk scores. The AVA and the mean PG achieved at the end of the procedure were well maintained at 2 year follow up and were statistically significant. The Supplementary Figures 4–12 in the manuscript depict data that has been collated and presented to demonstrate the patient profile, composition, and procedural outcomes.

Limitations of our study include its small sample size; however, we must recognise that TAVI is not a routinely common procedure in India. Therefore, the sample size of 70 provided helpful study results and future applications as the standard of therapy for bicuspid aortic stenosis patients. TAVI being the new procedure in India, the findings of our study can enthuse young interventionalists to pursue research in this area. Our study depicts results that can be expected in real-world clinical practice. All 68 patients at the end of 2 years remained stable, which itself is a testimony to the effectiveness and safety of TAVI in a bicuspid population.

**FIGURE 1:**
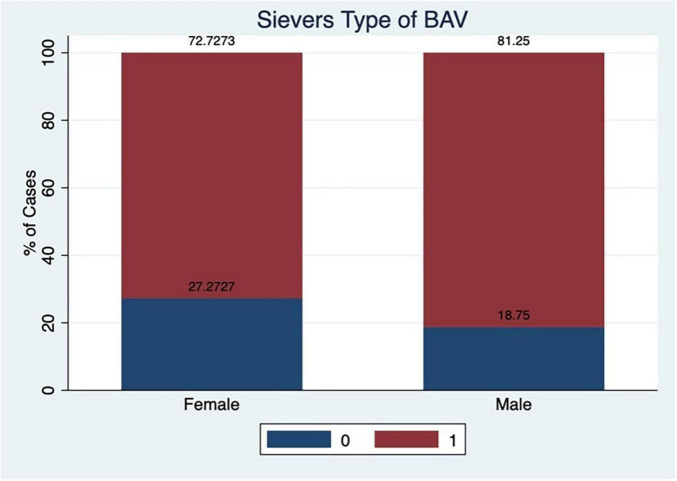
Pair wise comparison of mean pressure gradients over 2 years, statistically significant.

**FIGURE 2:**
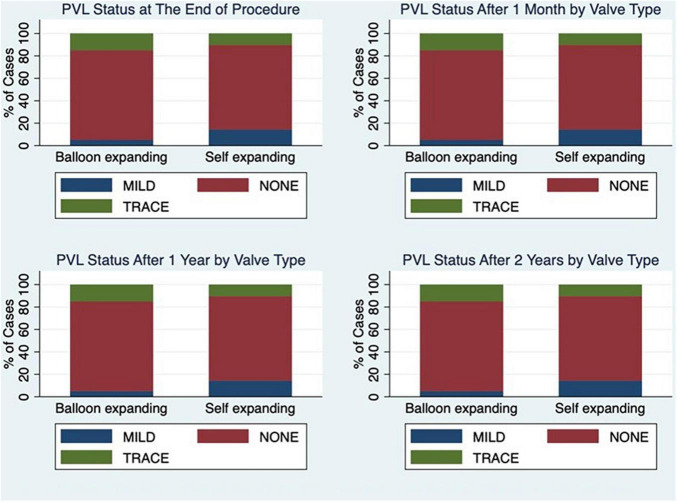
Pair wise comparison of average valve area over 2 years after TAVI.

**FIGURE 3:**
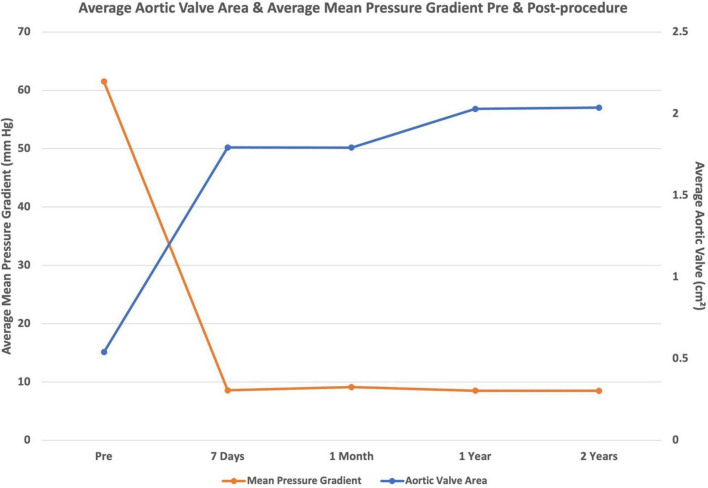
Mean pressure gradient and aortic valve area at 2 years after TAVI.

Statistical tests were applied to estimate significant differences between the pre and post procedure AVA and mean PG comparison groups, as depicted in Figures 1, 2. These show good haemodynamics at the end of 2 years, with an average valve area close to the magic number of 2 cm^2^ and a mean PG of less than 15 mmHg (Figure 3). More haemodynamic data and detailed longer follow up to 5 and 10 years, combined with CT imaging may throw light on early signs of structural degeneration and THVs failure. These aspects would be important for establishing this therapy for a young population who must lead an active life. Those with heavy calcium scores had a greater residual gradient and greater leak immediately after implantation and required post dilation to expand the frame. The peak-to-peak gradient reduced as did the leak to a mild degree. Sizing of the valve was done by the aforementioned algorithms and consideration of other anatomical factors. Adequate oversizing (5–10%) for BEV and (15–25%) oversizing for SEV were targetted. The risk of patient prosthesis mismatch was from an undersized valve as per the body surface area of the patient, which was also taken into account during valve size selection. If the aortic annulus was smaller in size, we preferred choosing a larger self-expanding valve over a smaller BEV. The size chosen was also not big enough to cause injury to the root.

The selection of the type of valve in our study was performed with some preferences of one over the other, e.g.: a BEV was preferred in the presence of horizontal aortic root and dilated ascending aorta. A BEV was avoided if the aortic annulus calcium extended to the LVOT. BEVs with large open struts were preferred if the coronaries ostia were at risk for occlusion.

Adequate predilation for every case was undertaken in our study. The balloon size for predilation was one size smaller or equal to the size of the minor axis of the aortic annulus diameter. This possibly opened the native valve adequately and prepared a good bed for implantation of the new valve with the least constraint, least gradient, and the least residual leak. This was the key factor in achieving the best haemdynamics, apart from the ideal selection of a particular valve size based on different anatomical measurements and considerations. It also helped provide an estimate of the appropriate size of valve. A shallower positioning and supra-annular implantation were aimed in every SEV case and a 90–10 to 70–30 depth implantation was aimed for in the BEV cases.

Whenever required post dilation was undertaken with an appropriate size of non-compliant balloon (perimeter or area derived diameter) that resulted in eliminating the residual gradient and leak. Post dilation was performed if there was a residual gradient >15 mmHg or a significant PVL was seen due to an under expanded valve. The PVL was moderate to severe in 24.28% of cases immediately after deployment of the valve and was reduced to trace or mild in most of the patients at the end of the procedure, which was maintained at a follow up of 2 years irrespective of the type of valve used. There was no moderate or severe PVL at 2 year follow up. A second valve in valve was implanted for two cases of severe PVL immediately at end of the procedure due to the final deeper implantation of the first valve. Longer years of follow up of the mild PVL would be needed to assess its progression and clinical impact. In our study mal apposition or under expansion of the frame, constraint in the frame due to calcium rocks was the cause of PVL. An under sized valve implantation was not the cause of PVL in the study. Newer generation valves with external skirts also contributed to reducing the PVL to a minimum even in the presence of calcium chunks.

**The PPI** rate was higher in the SEV group as compared to the BEV group by 30 days. The possible reasons were a final deeper implantation and pre-existing conduction block. PPI was not needed in any patient in either group at 1- or 2-year period follow up. CHB needing PPI is unacceptable for the young population and thus needs more emphasis on shallow but safe depth of implantation, measuring membranous septum length on CT, and positioning it above that level at high pacing rates during deployment to avoid the deep diving of the valve, maintaining the forward push on the wire to prevent diving deep during deployment, recapturing if you have gone deep and very recently cuspal overlap technique has also been used for bicuspid valve implantation.

In our study, disabling stroke happened to none of the patients but 10% of patients had periprocedural non-disabling stroke, which was seen more with BEVs, possibly because of predilation and postdilation in the setting of heavily calcified valves resulting in showers of microemboli. A dedicated cerebral protection device for TAVI is not yet available in the country and so is not used in routine practice. We used one spider filter and an Emboshield device on our patient who had the presence of mobile healed vegetation or atheromatous/calcified mobile mass attached to the leaflet. The patient had no stroke and the debris was trapped. Possible reasons for stroke appear to be the embolising calcium particles from the practice of mandatory predilation. Valve repositioning and repeated recapturing of the self-expanding valves and the post dilation were also factors responsible for the occurrence of stroke. Stroke in the young population is very much an unacceptable complication, as it could be disastrous and ruin their lives. Stroke, even when non-disabling, is unacceptable for a young population and the importance of cerebral protection in the bicuspid population becomes more important. Secondly, the role of routine anticoagulation for 3 months to 1 year also needs to be studied to avoid thrombosis of the microparticles of calcium embolised into cerebral circulation and causing delayed strokes in the first week or by 30 days of the implantation or potentially showers of emboli from a silent thrombosis of the tissue of the new valve.

Symptomatic NYHA class improvement by at least one or more functional classes was seen in 100% of patients. There was an improvement in the class of symptoms for the LV dysfunction subset of patients as well. The LV systolic function was mostly near normal. Those with severe LV dysfunction also had improved ejection fraction by 5–15% but the size of this subset of patients was small and statistical analysis was not possible. A study purely evaluating poor LV systolic function cases is needed to examine why some patients improved only marginally (possibly due to factors like irreversible fibrosis or elements of some kind of cardiomyopathy), which prevented the heart function from improving to near normal. Moreover, we require studies dedicated to looking at readmission rates from heart failure and quality of life indices in the presence of non-improvement of LV systolic function after new valve implantation. Some indices need to be established for suggesting which subset of LV dysfunction patients would improve and who would not improve.

## Conclusion

The Indian experience of TAVI in the BAV patient population is quite similar to that described in other literature from across the world. The BAV is present in a fairly high percentage of the Indian population of aortic stenosis patients. TAVI is extending fast to this subset of severe AS patients but important aspects of the success of this therapy will be to take into account sizing and implantation, freedom from PVL, pacemaker implantation, and stroke. Coronary safety and ease of access in future are connected to its 10–15 years of durability and freedom from patient prosthesis mismatch. Moreover, our findings indicate that good and sustained haemodynamics with an aortic valve orifice area of around 2 cm^2^ should be given to the young population. Once most of these are achieved, the therapy will be used more and large randomised studies will be needed. Meticulous understanding and analysis of CT scan imaging may help to exclude certain sets of these BAV anatomies who are labelled unsuitable for TAVI and should be offered surgery. The suitability of this therapy for very young patients in their 20–50 s is an unanswered question because they may need more than one valve replacement procedure during their life, depending on which therapy is chosen as their index procedure.

## Data Availability Statement

The original contributions presented in this study are included in the article/Supplementary Material, further inquiries can be directed to the corresponding author.

## Ethics Statement

Ethical review and approval was not required for the study on human participants in accordance with the local legislation and institutional requirements. Written informed consent for participation was not required for this study in accordance with the national legislation and the institutional requirements.

## Author Contributions

VK, GS, VPS, and VR collated the data from their centres and done the analysis of the entire data. VK wrote the manuscript and referencing. AS reviewed the content of the manuscript and incorporated his needful suggestions and inputs in the manuscript. All authors contributed to the article and approved the submitted version.

## Conflict of Interest

AS was Proctor for TAVI and received consulting fee from Meril Lifesciences and Medtronic. The remaining authors declare that the research was conducted in the absence of any commercial or financial relationships that could be construed as a potential conflict of interest.

## Publisher’s Note

All claims expressed in this article are solely those of the authors and do not necessarily represent those of their affiliated organizations, or those of the publisher, the editors and the reviewers. Any product that may be evaluated in this article, or claim that may be made by its manufacturer, is not guaranteed or endorsed by the publisher.
