# Supplementary figures and images for: Transcatheter Aortic Valve Implantation for Severe Bicuspid Aortic Stenosis – 2 Years Follow up Experience From India
Source: Front Cardiovasc Med. 2022 Jul 28;9:817705. doi: 10.3389/fcvm.2022.817705 (PMC9369256; doi:10.3389/fcvm.2022.817705)

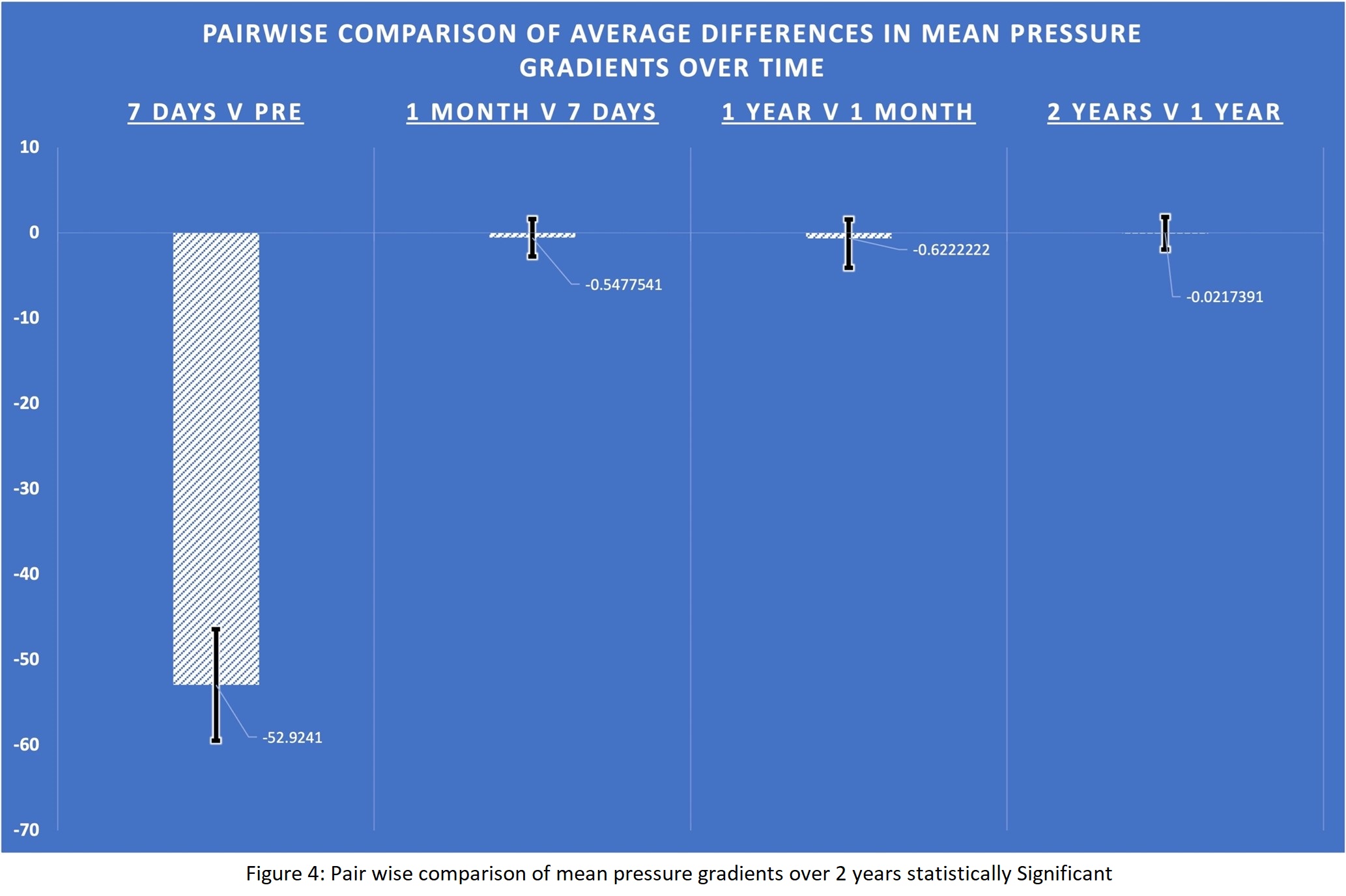

Supplement: Supplementary file 1 [file Image_1.jpg]

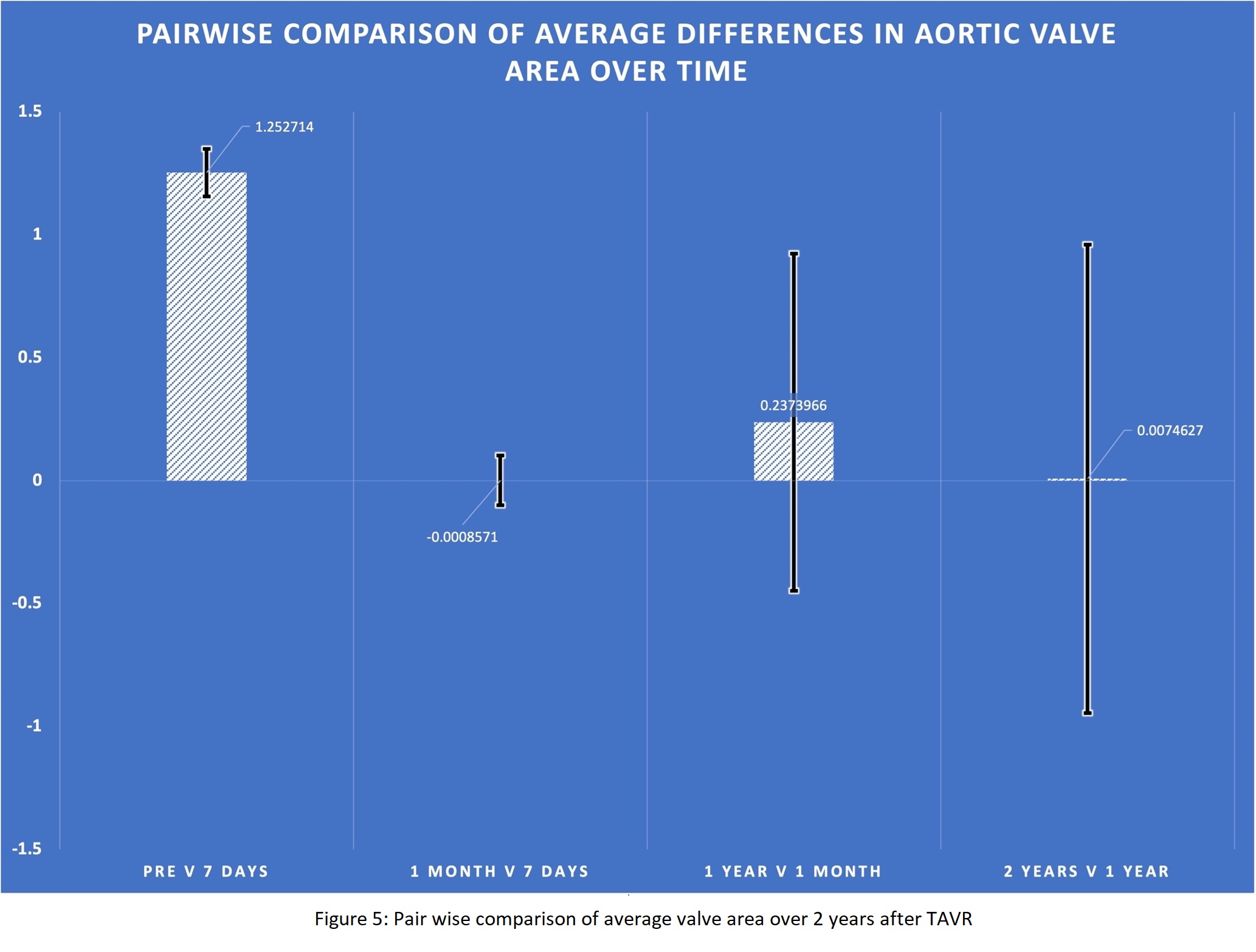

Supplement: Supplementary file 2 [file Image_2.jpg]

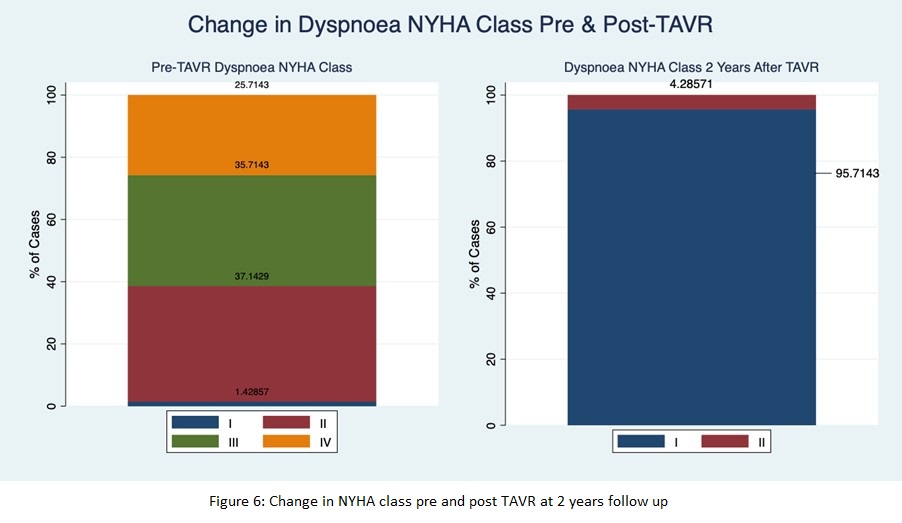

Supplement: Supplementary file 3 [file Image_3.jpg]

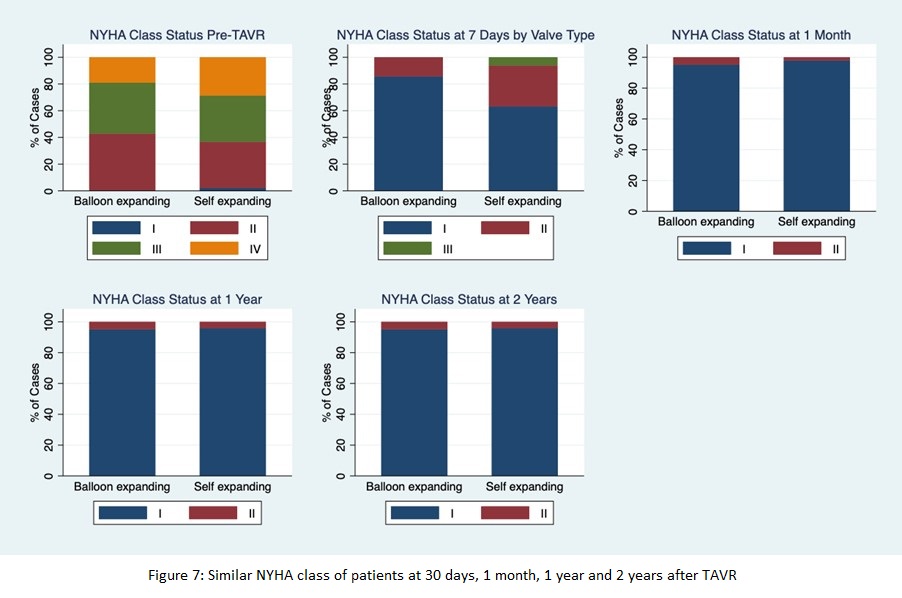

Supplement: Supplementary file 4 [file Image_4.jpg]

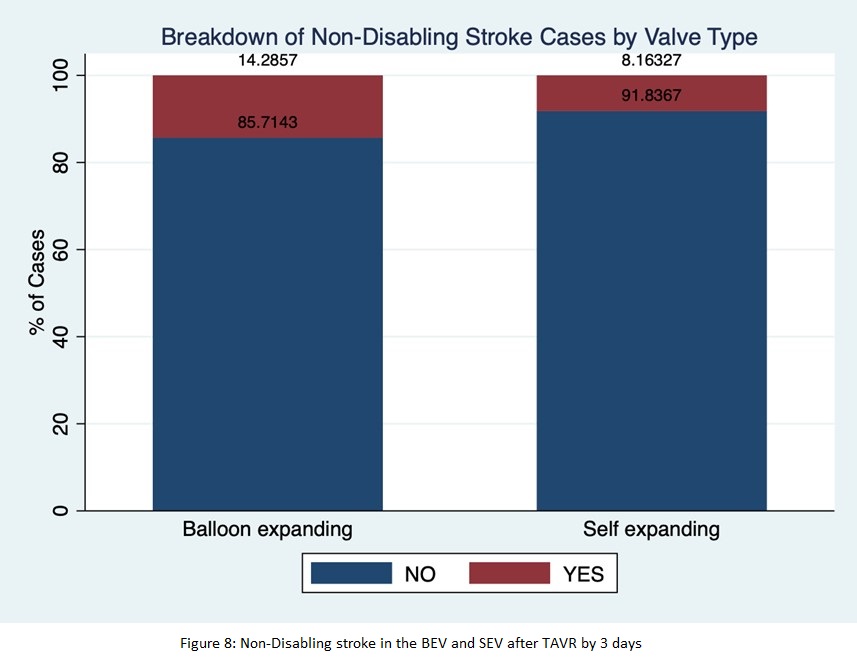

Supplement: Supplementary file 5 [file Image_5.jpg]

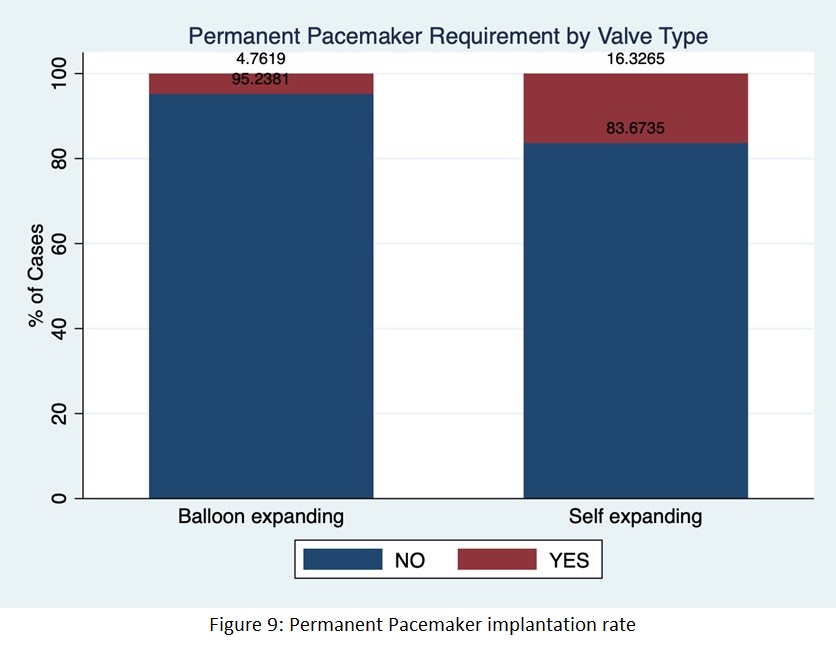

Supplement: Supplementary file 6 [file Image_6.jpg]

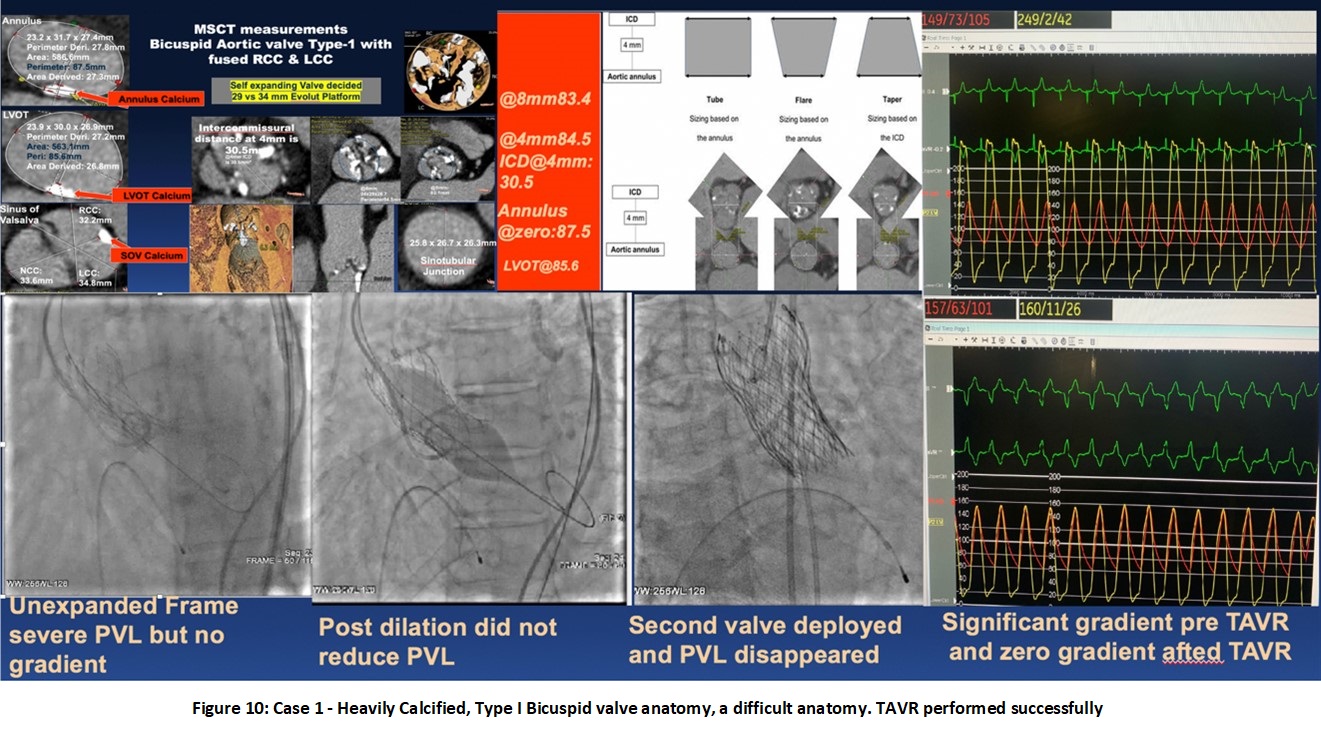

Supplement: Supplementary file 7 [file Image_7.jpg]

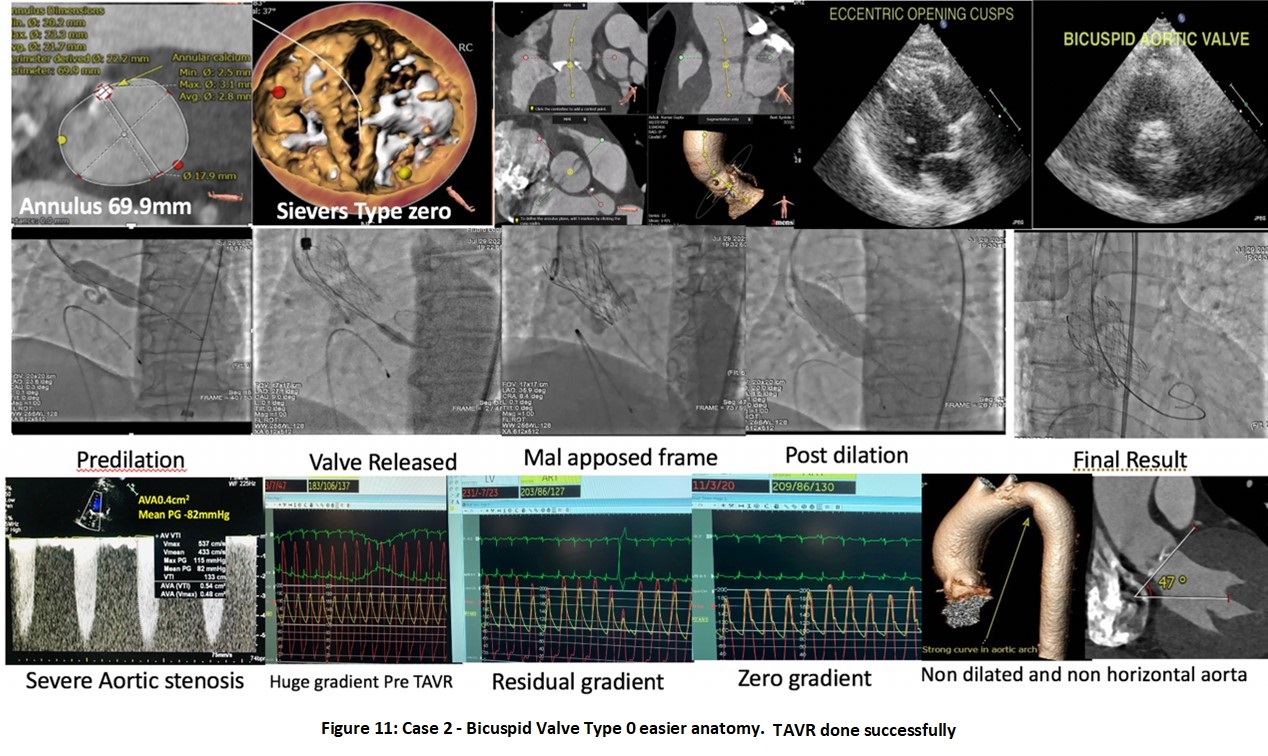

Supplement: Supplementary file 8 [file Image_8.jpg]

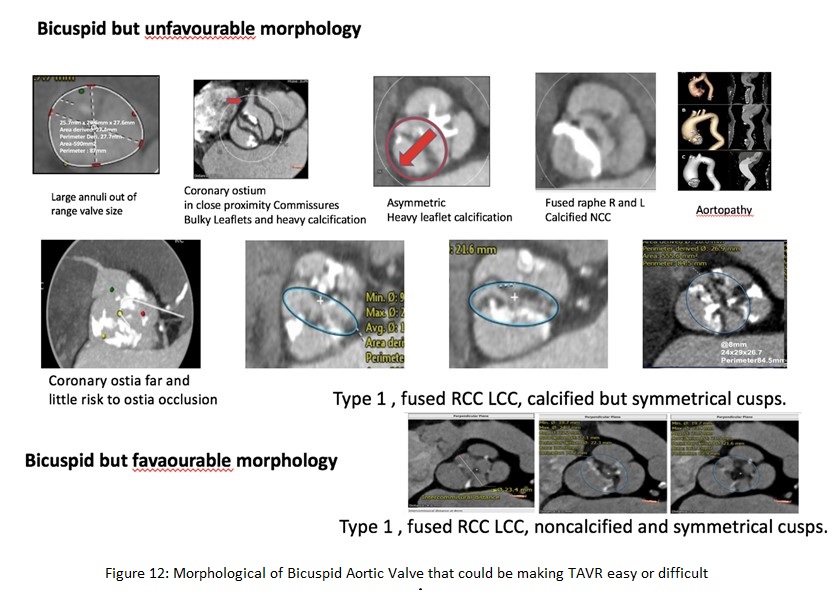

Supplement: Supplementary file 9 [file Image_9.jpg]
